# A diffusion tensor imaging study of brain microstructural changes related to religion and spirituality in families at high risk for depression

**DOI:** 10.1002/brb3.1209

**Published:** 2019-01-15

**Authors:** Xuzhou Li, Myrna Weissman, Ardesheer Talati, Connie Svob, Priya Wickramaratne, Jonathan Posner, Dongrong Xu

**Affiliations:** ^1^ East China Normal University Shanghai China; ^2^ Department of Psychiatry Columbia University New York New York; ^3^ New York State Psychiatry Institute New York New York

**Keywords:** diffusion tensor imaging, major depressive disorder, microstructural abnormality, religion, spirituality, voxel‐wise analysis

## Abstract

**Introduction:**

Previously in a three‐generation study of families at high risk for depression, we found that belief in the importance of religion/spirituality (R/S) was associated with thicker cortex in bilateral parietal and occipital regions. In the same sample using functional magnetic resonance imaging and electroencephalograph (EEG), we found that offspring at high familial risk had thinner cortices, increased default mode network connectivity, and reduced EEG power. These group differences were significantly diminished in offspring at high risk who reported high importance of R/S beliefs, suggesting a protective effect.

**Methods:**

This study extends previous work examining brain microstructural differences associated with risk for major depressive disorder (MDD) and tests whether these are normalized in at‐risk offspring who report high importance of R/S beliefs. Diffusion tensor imaging (DTI) data were selected from 99 2nd and 3rd generation offspring of 1st generation depressed (high‐risk, HR) or nondepressed (low‐risk, LR) parents. Whole‐brain and region‐of‐interest analyses were performed, using ellipsoidal area ratio (EAR, an alternative diffusion anisotropy index comparable to fractional anisotropy). We examined microstructural differences associated with familial risk for depression within the groups of high and low importance of R/S beliefs (HI, LI).

**Results:**

In the LI group, HR individuals showed significantly decreased EAR in white matter regions neighboring the precuneus, superior parietal lobe, superior and middle frontal gyrus, and bilateral insula, supplementary motor area, and postcentral gyrus. In the HI group, HR individuals showed reduced EAR in white matter surrounding the left superior, and middle frontal gyrus, left superior parietal lobule, and right supplementary motor area. Microstructural differences associated with familial risk for depression in precuneus, frontal lobe, and temporal lobe were nonsignificant or less significant in the HI group.

**Conclusion:**

R/S beliefs may affect microstructure in brain regions associated with R/S, potentially conferring resilience to depression among HR individuals.

## 1 INTRODUCTION

Major depressive disorder (MDD) is a common but debilitating psychiatric disorder. Parental depression is among the most consistently replicated risk factors for MDD, with a number of studies showing that having one or more depressed parents increases the risk of depression in the offspring twofold to fourfold (Hammen, Burge, Burney, & Adrian, 1990; Klein, Lewinsohn, Seeley, & Rohde, 2001; Lieb et al., 2000; Weissman, Berry et al., 2016; Weissman, Wickramaratne et al., 2016; Weissman et al., 2006). However, only some offspring exposed to parental depression go on to develop symptoms; conversely, the disorder also occurs in individuals with no family history, suggesting that other biological and environmental factors must also play an important role (Peterson et al., 2009).

One such factor that has only recently begun to receive attention is belief in the importance of religiosity/spirituality (R/S) (Miller et al., 2014). Religious belief and behavior are considered by some theorists as complex brain‐based phenomena that may have emerged in our species along with novel cognitive processes for social cognition and successfully engaged fundamental cognitive mechanisms, such as memory (Bloch, 2008; Boyer, 2008; Boyer & Bergstrom, 2008). Several studies have documented a protective effect of religiosity on depression (Abdel‐Khalek, 2006; Azari et al., 2001; Fox et al., 2014; Harris et al., 2009; Kapogiannis, Barbey, Su, Zamboni et al., 2009; Lazar et al., 2005; Miller et al., 2014, 2012).

We previously documented that among adults at high familial risk for depression (by virtue of having one or more biological parents with the disorder), R/S demonstrated a protective effect on those who reported that R/S was highly important to them from experiencing a recurrence of MDD (Miller et al., 2012). Attendance at religious services and religious denomination in contrast was not associated with lower rates of depression (Miller et al., 2014), suggesting that *intrinsic* R/S beliefs, rather than *externalized *components of religiosity, were conferring the protective effect (Allport & Ross, 1967).

We next tested the neurobiological underpinnings of R/S in families at high and low risk for depression. We found that belief in the importance of R/S was associated with *thicker* cortices in bilateral parietal and occipital regions, particularly the cuneus and precuneus (Liu et al., 2017; Miller et al., 2014). As we had previously reported cortical *thinning *in these regions as a stable biomarker for depression risk (Hao et al., 2017; Peterson et al., 2009), we hypothesized that the thicker cortices in those reporting high importance of R/S beliefs may serve as a compensatory or protective mechanism. In subsequent studies on the same participants, we found increased default mode network (DMN) connectivity (Posner et al., 2015), and greater EEG alpha among individuals at high (compared to low) familial risk for depression (Tenke et al., 2013, 2017). However, within the subgroup of individuals at high risk for depression, those reporting that R/S was highly important to them had reduced DMN connectivity (Svob, Wang, Weissman, Wickramaratne, & Posner, 2016) and greater EEG alpha (Tenke et al., 2013, 2017). In other words, brain differences associated with importance of R/S beliefs were the opposite of those associated with disease risk, suggesting that R/S may provide a compensatory mechanism or buffering effect.

Despite these studies, little is known about brain connectivity in the context of religious experience. To better understanding the neurobiological correlates of R/S belief, more advanced MRI measures can be useful as they become available. In this regard, diffusion tensor imaging (DTI) is able to distinguish between cortical thickness and interconnectivity by measuring, specifically, white matter integrity. This approach may give us a better picture of the neurobiological processes underlying R/S beliefs and its potentially protective effects against depression. Only one DTI study to our knowledge has used DTI to examine white matter integrity in relation to R/S beliefs (Xu, McClintock, Balodis, Miller, & Potenza, 2018). The study found that openness to changing religious views was associated with greater white matter integrity in the genu of the corpus callosum. Although the findings are compelling, the sample was small and was restricted to healthy adults.

In the present study, we extend our prior work by examining structural connectivity, based on DTI data. The purpose is to identify differences in brain microstructure associated with R/S beliefs that may be relevant to conferring protective effects to offspring at high familial risk for depression. We expected that individuals at high as compared to low familial risk for depression would show significant white matter microstructural changes in white matter tracts adjacent to brain regions implicated in familial risk for MDD. Furthermore, if protective effects of R/S are mediated through brain microstructural differences, microstructural differences between high‐ and low‐risk offspring should be diminished in those rating R/S as highly important.

## 2 MATERIALS AND METHODS

The cohort from which this sample is drawn has been previously described in several publications (Miller et al., 2014, 2012; Peterson et al., 2009; Weissman, Berry et al., 2016; Weissman, Wickramaratne et al., 2016; Weissman et al., 2006). Briefly, the study began in 1982 with the simultaneous recruitment of two groups of probands (Generation 1). One group, with major depression, was selected from an outpatient psychiatric clinic for the treatment of mood disorders in the New Haven, CT, area and was required to have moderate to severe MDD with impairment in functioning. The nondepressed probands were selected from the same community and were required to have no lifetime history of psychiatric illness, based on several interviews. All probands were of European, and primarily Southern Italian, ancestry. Their biological children (G2) and, subsequently, grandchildren (G3) were followed prospectively over time. The offspring of the depressed probands formed the “high‐risk” group, and those of the nondepressed probands, the “low risk” group (Weissman, Berry et al., 2016; Weissman, Wickramaratne et al., 2016; Weissman et al., 2006). There were six longitudinal waves of assessments including at year 0 (baseline/recruitment) and approximately 2, 10, 20, 25, and 30 years thereafter.

Diagnostic assessments were completed at each wave using the Schedule for Affective Disorders and Schizophrenia–Lifetime Version (Koenig, King, & Carson, 2012; McCullough & Willoughby, 2009). Each family member was interviewed independently and blinded to the clinical or R/S status of other family members, by trained doctoral‐ and masters‐level mental health professionals (reliability, which was high, has been documented elsewhere (Weissman et al., 2006; Weissman, Berry et al., 2016; Weissman, Wickramaratne et al., 2016)).

### 2.1 Participants and data acquisition

DTI data were collected at time 30 (T30) only. A total of 122 participants from G2 and G3 were recruited for this study, and finally 99 entered the present analysis (see detail below). All procedures were approved by the institutional review board at New York State Psychiatric Institute/Columbia University, and informed consent was obtained. Data on the importance of R/S beliefs were also acquired at T30, concurrent with the DTI data for modeling whether R/S detected microstructural differences in the brain.

DTI data were acquired on a 3 T, whole body, GE Signa HDx MRI scanner (GE Medical Systems, Milwaukee, WI) with an 8‐channel head coil (InVivo Corporation, Orlando, FL). A single‐shot echo‐planer imaging (EPI) pulse sequence was used and collected diffusion‐weighted images along 25 noncollinear diffusion gradients (b = 1,000 s/mm^2^) and three volumes of nondiffusion‐weighted images (b = 0 s/mm^2^, baseline images). The DTI acquisition was in contiguous axial planes with whole‐brain coverage using parameters as follows: TR = 17,000 ms; TE = 78.2 ms; NEX = 2; field of view = 24 × 24 cm^2^; acquisition matrix = 256 × 256; slice thickness = 2.5 mm without inter‐slice gaps; and number of slices: 58–71, as the participants varied from children to adults.

### 2.2 Religion and spirituality measures

The assessment of religiosity was determined by asking the following three questions, which are the most frequently used in research on religion and health (Koenig et al., 2012; Larson & Larson, 2003; Miller et al., 2014): (a) “How important to you is religion or spirituality?” The responses to the importance (Question 1) dichotomized as not defined (=1), high (=2), or low (≥3) importance, respectively, consistent with previous studies; (b) “How often, if at all, do you attend church, synagogue, or other religious or spiritual services?” with a response ranging four categories from never to once a week or more; and (c) “How would you describe your current religious beliefs? Is there a particular denomination or religious organization that you are part of?” The response to this question was not used in the present study because most of the subjects fell into two groups. We focus on the importance variable given that our previous work has identified that importance, but not other questions related to R/S confer a protective effect against depressive illness. Of our analytic sample of 99 individuals, 22 participants reported high importance and 77 reported low or moderate importance. Level of personal importance of religion or spirituality did not differ by risk groups.

### 2.3 Data preprocessing

We checked a total of 122 (65 HR + 57 LR) DTI datasets both visually and quantitatively and removed datasets with apparent artifact, head motion (>2 mm or 1 degree of rotation), or poor image quality. The steps of preprocessing were as follows: (a) Visual and quantitative inspection of the DWI data, and remove data of bad quality, following the criteria just mentioned; (b) Eddy current correction: This step was performed using the FMRIB Software Library (FSL 6; http://www.fmrib.ox.ac.uk/fsl) and our own method (Liu et al., 2013)), including registering all volumes of diffusion‐weighted imaging (DWI) data to the baseline images; (c) Calculated a binary mask for the b0 images, and removed nonbrain tissues; (d) Tensor estimation: using the software tools developed in‐house, which was based on the data that were corrected; (e) Spatial normalization: One participant from the low‐risk nondepressive group was selected as a template, and then deformation fields were calculated by coregistering each participant's baseline image to the template (the template was selected by an anatomy expert, who reviewed all the image data and selected the one that appeared visually high quality and with normal structures); (f) DTI warping, by applying the deformation field for each participant to be normalized to the template space (Xu, Hao, Bansal, Plessen, & Peterson, 2008; Xu, Mori, Shen, Zijl, & Davatzikos, 2003); and (g) Ellipsoidal area ratio (EAR), a diffusion index very similar to fractional anisotropy (FA) but more sensitive to changes in brain than FA (Afzali & Soltanian‐Zadeh, 2010; Kang, Herron, & Woods, 2010; Xu et al., 2009) was calculated voxel‐wise. After the seven steps of preprocessing, we further excluded DTI datasets that failed to be coregistered with adequate accuracy (thresholded at 2 mm) due to invisible image noise. Finally, we got 99 sets of usable DTI data (53 HR and 46 LR) with R/S data for voxel‐based analyses at T30.

### 2.4 Ellipsoidal area ratio

Ellipsoidal area ratio is an anisotropy diffusion index similar to fractional anisotropy (FA), ranging between 0 and 1, with 0 indicating isotropic diffusion and 1 totally isotropic. We selected EAR as it is more sensitive than FA in detecting white matter abnormalities in patients with widespread diffuse axonal injury, and it has improved signal‐to‐noise ratio relative to FA, suggesting superiority for quantifying alterations in white matter structure (Afzali & Soltanian‐Zadeh, 2010; Kang et al., 2010). EAR has been widely used in many applications studying various populations and diseases (Afzali & Soltanian‐Zadeh, 2010; Ahmed Sid, Abed‐Meraim, Harba, & Oulebsir‐Boumghar, 2017; Gong, 2013; Holstein, Smith, & Paris, 2016; Sun et al., 2012; Zhou et al., 2011). Just like the FA measure, increased EAR typically suggests higher integrity in white matter, whereas decreased EAR the opposite including more complex or messy situations such as increased fiber crossing.

### 2.5 Voxel‐wise analysis

The voxel‐wise analyses of EAR maps were performed using Statistical Parametric Mapping (SPM8, http://www.fil.ion.ucl.ac.uk/spm), MATLAB R2014a, after the data were all normalized to the selected template space, as previously described. In order to label the brain regions, we mapped our population‐specific template to the Montreal Neurological Institute (MNI) space based on an EPI template and then generated the transformation matrix between the previous template and the MNI template. Consequently, all the results generated from our analyses can be properly labeled and identified by checking their correspondence with the MNI template. We smoothed all the EAR maps with a full‐width at half maximum of the Gaussian smoothing kernel of 8 mm. Afterward, a linear regression adjusted with covariates (age, sex, handedness, etc.) was set up for comparisons based on the smoothed EAR maps. To examine the effects of religion importance for depression on the individuals, we used two‐sample *t* tests.

To investigate how the importance of R/S beliefs may affect the development of MDD in people at different familial risk status, we conducted three analyses: (a) Compared high importance (HI) versus low importance (LI) (*N* = 10:43) within the HR group, to detect the associations between the importance of R/S beliefs and brain microstructure within the individuals at high familial risk for MDD; (b) Compared HI versus LI (*N* = 12:34) within the low‐risk group, to detect the associations between the importance of R/S beliefs and brain microstructure within the individuals at low familial risk for MDD; (c) Analyze the difference in brain microstructure between HR and LR within the HI group and that within the LI group (*N* = 10:12 vs. 43:34), to examine the effect of R/S beliefs on the difference related to familial risk for developing MDD. In the analyses, the threshold was set at a cutoff at *p* < 0.001 (uncorrected) combined with a minimal cluster size of 100 voxels to be considered as significant difference. The combined use of *p*‐value and the cluster size therefore served as a stricter threshold to identify statistical significance, thereby reducing the possibility of false discovery.

## 3 RESULTS

### 3.1 Demographics

Demographic characteristics of the samples document no significant differences by the importance of R/S beliefs (Table 1). Data from 99 participants (mean age and standard deviation at scanning: 33.45 ± 13.48 years; age range: 10–59 years; 45 males and 54 females) with usable DTI and R/S data are used in this study. The 99 persons did not differ statistically significantly from the full dataset on key variables including risk status, R/S, handedness, sex, and age. Among the participants, 54% were women, 37% were married or remarried, 41% were single, 7% were separated or divorced, and 14% did not report marital status. 37% of them had higher education than high school, 48% had a high school diploma or general educational development certificate even had not graduated high school, and 14% did not report educational attainment. The annual incomes of 23% participants were below $20,000, 22% between $20,000 and $39,999, and 32% above $40,000, and 22% of them did not report. The handedness summary showed that 89.9% participants were right‐handed and 10.1% were‐left handed. The distribution of religious denomination was as follows: 59% Catholic, 11% Protestant, 9% espoused personal R/S beliefs not affiliated with any institutionalized religion, 16% other religions, and 5% gave no response.

**Table 1:** Demographic, religious, and clinical characteristics in high‐ and low‐risk groups. All the data were collected at T30

Demographic of the high‐ and low‐risk groups		
Characteristic	High risk	Low risk
Age, Mean (*SD*)^a^	34.03 (14.22)	31.07 (14.56)

	*N* (%)	*N* (%)
Sex^b^		
Female	28 (28.2%)	26 (26.2%)
Male	25 (25.2%)	20 (20.2%)
Religious importance		
High importance (=2)	10 (10.1%)	12 (12.1%)
Low Importance (>2)	43 (43.4%)	34 (34.2%)
Annual incomes ($)		
<20,000 (low)	14 (14.1%)	9 (9.1%)
20,000–39,999 (medium)	9 (9.1%)	13 (13.1%)
>40,000 (high)	18 (18.1%)	14 (14.1%)
No information	12 (12.1%)	10 (10.1%)
Marital status		
Single	19 (7.1%)	22 (32.3%)
Married or remarried	20 (31.3%)	17 (8.1%)
Separated or divorced	5 (2.1%)	2 (4.1%)
No information	9 (13.1%)	5 (2.1%)
Education level		
<=High School	25 (25.2%)	23 (23.2%)
>High School	19 (19.2%)	18 (18.2%)
No information	9 (9.1%)	5 (5.1%)
Handedness		
Right	50 (50.5%)	39 (39.4%)
Left	3 (3.0%)	7 (7.1%)
Religious denomination		
Catholic	25 (25.3%)	33 (33.2%)
Protestant	8 (8.1%)	3 (3.1%)
Personal Spirituality	6 (6.1%)	3 (3.1%)
Agnostic/Atheist/Other	12 (12.1%)	4 (4.1%)
No information	2 (2.1%)	3 (3.1%)

a *F* test shows no significant difference in age (*p* = 0.325) in different family risk status.

b Chi‐square test shows no significant difference.

### 3.2 Effects of importance of R/S beliefs on brain microstructure in offspring at high and low risk for depression

**Figure 1:**
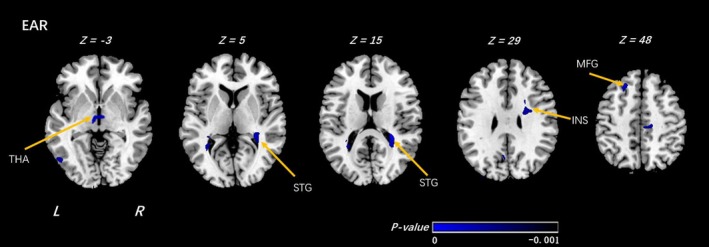
Voxel‐wise analyses of EAR maps between the HR_HI and HR_LI groups. In the comparison under high family risk (HR) group of individuals at high religion importance (HI) and individuals at low religion importance (LI), significantly decreased EAR was detected by voxel‐wise analyses within the individuals at high religion importance. The threshold was set as *p* < 0.001 (uncorrected) and a minimum cluster size = 100 voxels. Arabic numeral indicates the location of each axial section within the standard reference space of MNI. Color scale represents the *p*‐values. L = left, R = right. THA: thalamus; STG: superior temporal gyrus; INS: insula; MFG: middle frontal gyrus. The sign of *p*‐values indicates the direction of the significance: “–” for LI > HI

**Figure 2:**
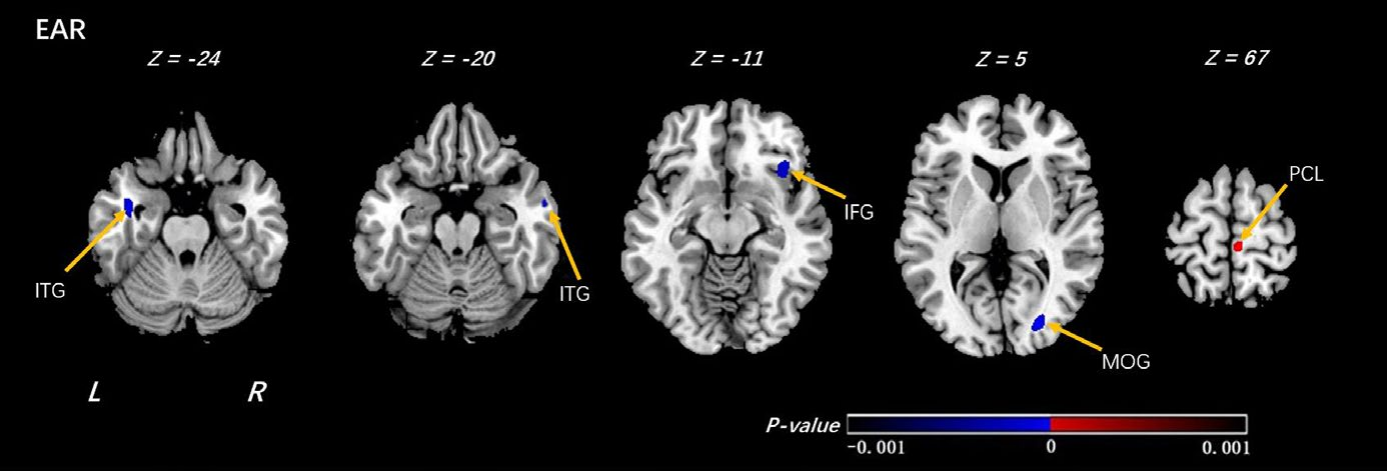
Voxel‐wise analyses of EAR map between the LR_HI and LR_LI groups. In the comparison under low family risk (LR) group of individuals at high religion importance (HI) and individuals at low religion importance (LI), significantly decreased and increased EAR were detected by voxel‐wise analyses within the individuals at high religion importance. The threshold was set as *p* < 0.001 (uncorrected) and a minimum cluster size = 100 voxels. Arabic numeral indicates the location of each axial section within the standard reference space of MNI. Color scale represents the *p*‐values. L = left, R = right. ITG: inferior temporal gyrus; IFG: inferior frontal gyrus; MOG: middle occipital gyrus; PCL: paracentral lobule. The sign of *p*‐values indicates the direction of the significance: “+” for HI > LI and “–” for LI > HI

Within offspring at high familial risk for depression, compared with those who reported low importance of R/S beliefs, individuals who reported high importance of R/S beliefs showed decreased EAR in white matter in bilateral forebrain adjacent to the bilateral thalamus, right insula and left middle frontal gyrus(MFG), and right superior temporal gyrus (Figure 1,Table 2). Among offspring at low risk, high importance of R/S beliefs was associated with decreased EAR in white matter neighboring the bilateral inferior temporal gyrus (ITG), right inferior frontal gyrus (IFG), and middle occipital gyrus (MOG). In contrast, increased EAR was detected in white matter near the right paracentral lobule (PCL) (Figure 2,Table 3).

**Table 2:** Voxel‐wise analyses of EAR maps between the HI and LI groups (under high family risk group)

High family risk group (high religion importance (HI)_VS_Low Religion Importance (LI))								
Clusters	Regions	R/L	MNI Coordinates			*T*‐value	Cluster size	Increase or decrease
			X	Y	Z			
EAR								
Thalamus	Thalamus_L (aal) Thalamus_R (aal)		−6	−10	−3	−3.4672	368	↓
Temporal Lobe	Superior Temporal Gyrus Transverse Temporal Gyrus	R	39	−38	15	−7.0298	1,584	↓
Frontal Lobe	Insula Extranuclear	R	31	7	15	−5.0186	1,227	↓
Frontal Lobe	Middle Frontal Gyrus	L	−21	29	49	−5.4561	287	↓

Note

Shown in the table are the significant differences when the individuals at high religion importance (HI) were compared with the individuals at low religion importance (LI) under high family risk group, with a design matrix of [1 − 1]. The threshold was set at a combined cutoff value of *p* < 0.001 (uncorrected) and a minimal cluster size of 100 voxels.

EAR, ellipsoidal area ratio; VS, versus; R/L, right or left; “↓” means decrease.

**Table 3:** Voxel‐wise analyses of EAR maps between the HI and LI groups (under low family risk group)

Low family risk group (High religion importance (HI)_VS_Low Religion Importance (LI))								
Clusters	Regions	R/L	MNI coordinates			*T*‐value	Cluster size	Increase or decrease
			X	Y	Z			
EAR								
Temporal Lobe	Middle Temporal Gyrus	L	−39	−4	−24	−4.3769	382	↓
Temporal Lobe	Inferior Temporal Gyrus Middle Temporal Gyrus	R	58	−4	−20	−4.3092	106	↓
Frontal Lobe	Inferior Frontal Gyrus	R	38	19	−8	−3.9347	578	↓
Occipital Lobe	Middle Occipital Gyrus Cuneus	R	27	−81	4	−4.8801	452	↓
Frontal Lobe	Paracentral_Lobule_R (aal) Medial Frontal Gyrus	R	5	−30	67	4.1831	342	↑

Note

Shown in the table are the significant differences when the individuals at high religion importance (HI) were compared with the individuals at low religion importance (LI) under low family risk group, with a design matrix of [1 − 1]. The threshold was set at a combined cutoff value of *p* < 0.001 (uncorrected) and a minimal cluster size of 100 voxels. EAR, ellipsoidal area ratio; VS, versus; R/L, right or left; “↑” means increase; “↓” means decrease.

### 3.3 Association between familial risk status and brain microstructure in offspring reporting high and low importance of religion/spirituality

**Figure 3:**
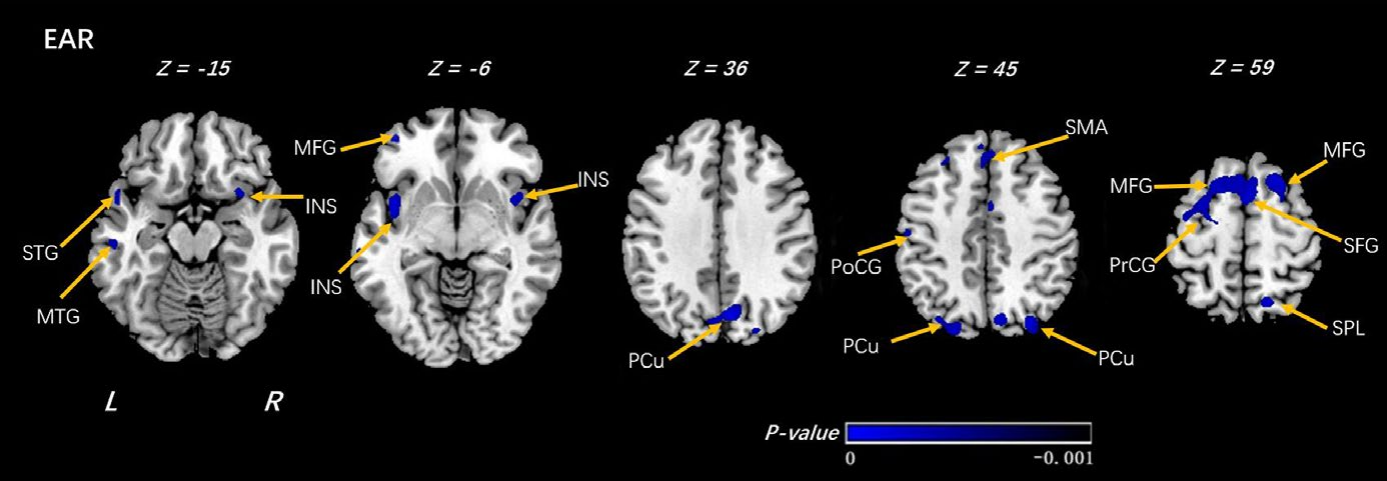
Voxel‐wise analyses of EAR maps between the LI_HR and LI_LR groups. In the comparison under low religion importance(LI) group of individuals at high risk (HR) and individuals at low risk (LR), significantly decreased EAR was detected by voxel‐wise analyses within the individuals at high risk. The threshold was set as *p* < 0.001 (uncorrected) and a minimum cluster size = 100 voxels. Arabic numeral indicates the location of each axial section within the standard reference space of MNI. Color scale represents the *p*‐values. L = left, R = right. PCu: precuneus; PoCG: postcentral gyrus; MTG: middle temporal gyrus; STG: superior temporal gyrus; SMA: supplementary motor area; SFG: superior frontal gyrus; MFG: middle frontal gyrus; INS: insula; PrCG: precentral gyrus. The sign of *p*‐values indicates the direction of the significance: “–” for LR > HR

We next compared differences associated with familial risk for depression among offspring who reported low and high importance of R/S beliefs, respectively. Among offspring who reported low importance of R/S beliefs, high familial risk was associated with decreased EAR in white matter neighboring a number of regions including the precuneus, superior parietal lobe, superior and middle frontal gyrus, and bilateral insula, supplementary motor area, and postcentral gyrus (Figure 3, Table 4).

**Table 4:** Voxel‐wise analyses of EAR maps between the HR and LR groups (under low religion importance group)

Low Religion Importance Group (High Family Risk (HR)_VS_Low Family Risk (LR))								
Clusters	Regions	R/L	MNI coordinates			*T*‐value	Cluster size	Increase or decrease
			X	Y	Z			
EAR								
Temporal Lobe	Middle Temporal Gyrus	R	64	−28	−9	−3.9158	186	↓
Frontal Lobe	Postcentral Gyrus	R	50	−17	52	−4.6567	463	↓
Parietal Lobe	Superior Parietal Lobule	R	16	−64	58	−4.8079	486	↓
Frontal Lobe	Superior Frontal Gyrus	R					7,782	
	Middle Frontal Gyrus	R					2036	
	Supp_Motor_Area_R(aal)	L				−5.1902	2071	
	Supp_Motor_Area_L(aal)	L					1926	
			1	18	54			↓
	Medial Frontal Gyrus	L					1515	
	Precentral Gyrus						874	
Parietal Lobe	Precuneus_R Precuneus_L	R L	9	−75	40	−5.7723	2,211	↓
Temporal Lobe	Middle Temporal Gyrus	L	−53	5	19	−4.5704	824	↓
Temporal Lobe	Superior Temporal Gyrus	L	−53	12	−10	−4.7824	239	↓
Frontal Lobe	Postcentral Gyrus	L	−50	−16	51	−5.7186	915	↓

Note

Shown in the table are the significant differences when the individuals at high risk of depression (HR) were compared with the individuals at low risk (LR), with a design matrix of [1 − 1]. The threshold was set at a combined cutoff value of *p* < 0.0001 (uncorrected) and a minimal cluster size of 100 voxels.

EAR, ellipsoidal area ratio; VS, versus; R/L, right or left; “↓” means decrease.

**Figure 4:**
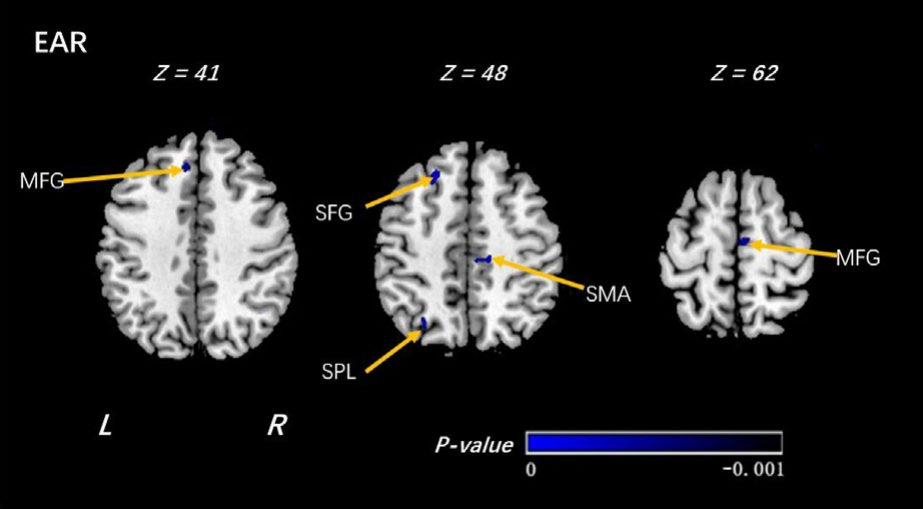
Voxel‐wise analyses of EAR maps between the HI_HR and HI_LR groups. In the comparison under high religion importance(HI) group of individuals at high risk (HR) and individuals at low risk (LR), significantly decreased EAR was detected by voxel‐wise analyses within the individuals at high risk. The threshold was set as *p* < 0.001 (uncorrected) and a minimum cluster size = 100 voxels. Arabic numeral indicates the location of each axial section within the standard reference space of MNI. Color scale represents the *p*‐values. L = left, R = right. SMA: supplementary motor area; SFG: superior frontal gyrus; MFG: middle frontal gyrus; SPL: superior parietal lobe. The sign of *p*‐values indicates the direction of the significance: “–” for LR > HR

Among those who reported high importance of R/S beliefs, offspring at high familial risk for depression had lower EAR in white matter surrounding the left superior, and middle frontal gyrus, left superior parietal lobule, and right supplementary motor area (Figure 4, Table 5).

**Table 5:** Voxel‐wise analyses of EAR maps between the HR and LR groups (under high religion importance group)

High Religion Importance Group (High Family Risk(HR)_VS_Low Family Risk(LR))								
Clusters	Regions	R/L	MNI Coordinates			*T*‐value	Cluster Size	Increase or Decrease
			X	Y	Z			
EAR								
Frontal Lobe	Supp_Motor_Area_R (aal)	R	15	−23	47	−4.6761	397	↓
Frontal Lobe	Medial Frontal Gyrus Supp_Motor_Area_R (aal)	L R	5	−15	62	−5.1346	264	↓
Parietal Lobe	Superior Parietal Lobule	L	−29	−66	47	−4.6081	244	↓
Frontal Lobe	Middle Frontal Gyrus	L	−7	35	41	−3.9742	138	↓
Frontal Lobe	Superior Frontal Gyrus Middle Frontal Gyrus	L	−23	27	48	−5.0176	391	↓

Note

Shown in the table are the significant differences when the individuals at high risk of depression (HR) were compared with the individuals at low risk (LR) under high religion importance group, with a design matrix of [1 − 1]. The threshold was set at a combined cutoff value of *p* < 0.001 (uncorrected) and a minimal cluster size of 100 voxels.

EAR, ellipsoidal area ratio; VS, versus; R/L, right or left.

**Figure 5:**
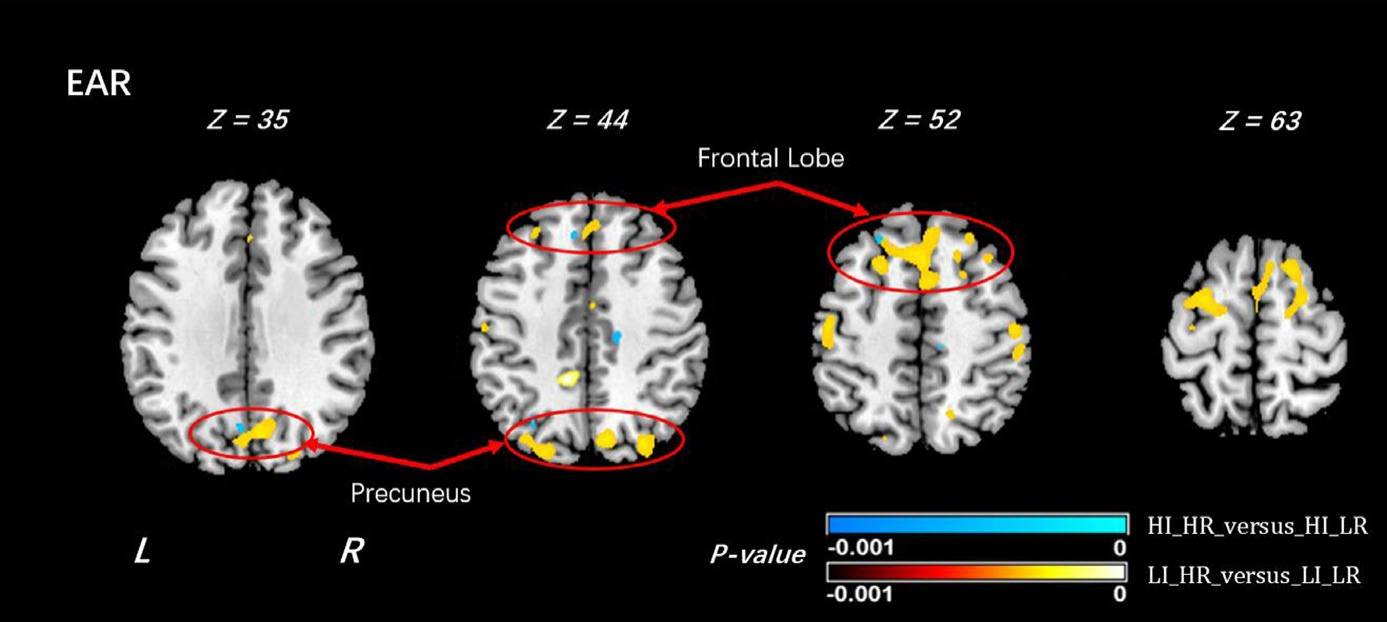
Difference of EAR results between the comparisons 3 and 4. In the comparison of the EAR results of HI_HR versus HI_LR shown in violet color, and the EAR results of LI_HR versus LI_LR shown in bright yellow and red. All the significance in areas close to precuneus regions disappeared in the results of HI_HR versus HI_LR (circled in red). The sign “‐” used in the *p*‐value colorbar is to indicate that the values were statistically smaller in the corresponding comparisons

Finally, we superimposed the findings in the high and low importance groups in order to highlight group differences. Risk differences in the low importance group are shown in yellow, those in the high importance group in blue. The result shows that most of the differences in risk status are in the low importance group (yellow parts in Figure 5), and these differences are diminished or disappear in the high importance group (Figure 5).

## 4 DISCUSSION

### 4.1 Associations between R/S Importance and brain microstructure

We investigated associations between brain microstructure and reported importance of R/S beliefs, in offspring at high and low familial risk for depression. In offspring at high familial risk, reduced microstructure strength was found in individuals who reported high importance of R/S beliefs in the thalamus, temporal lobe, insula, and middle frontal gyrus, when compared with those reported low importance of R/S beliefs. In offspring at low risk, individuals with high importance of R/S beliefs showed reduced EAR in the inferior frontal gyrus, inferior temporal gyrus, middle occipital gyrus, and increased EAR in the right paracentral lobule, when compared with those reported low importance of R/S beliefs.

Notably, the right inferior frontal gyrus only showed associations with R/S in offspring at low risk; conversely, decreased EAR in the white matter neighboring the right insula was only associated with R/S in offspring at high familial risk for depression. Previous studies have shown that brain abnormalities in the right insula are significantly correlated with R/S beliefs (Fox et al., 2014; Kapogiannis, Barbey, Su, Zamboni et al., 2009; Lazar et al., 2005; Wiebking et al., 2010). Given the key role the insula plays in emotional–cognitive integration (Augustine, 1996), deficits may imply a greater role of emotions in offspring at high risk for depression who deem R/S highly important. The findings thus agreed with the previous reports and also our expectations.

We next examined the associations between familial risk status for depression and white matter microstructure among offspring who reported either high or low importance of R/S beliefs. Among individuals reporting low importance of R/S beliefs, familial risk for depression was associated with lower EAR in bilateral frontal and parietal white matter, conforming to our previous studies showing gray matter abnormalities in these regions to confer vulnerability for familial depression. The findings also support previous work showing that importance of religiosity or spirituality was associated with thicker cortices in the left and right parietal and occipital regions, the right mesial frontal lobe of the right hemisphere, and the left precuneus (Miller et al., 2014). Importantly, the parietal and temporal lobe risk differences were not observed in the high importance group, and those within the frontal lobe were greatly attenuated.

The most notable difference was in the precuneus region (Figure 5). The absence of previously detected risk differences in individuals reporting high importance of R/S beliefs suggests a potential compensatory mechanism, with increased EAR providing a protective effect in offspring at high risk for depression. The precuneus has been implicated in both affective illness and in religious thought. Neuroanatomical studies have found higher religion/spirituality to be associated with thicker left precuneus (Miller et al., 2014; Peterson et al., 2009); functional studies similarly show greater activation when one is engaged in tasks of religiosity beliefs involving three psychological dimensions (God's perceived level of involvement, God's perceived emotion, and doctrinal/experiential religious knowledge) among individuals who report high religious beliefs (Kapogiannis, Barbey, Su, Krueger, & Grafman, 2009; Kapogiannis, Barbey, Su, Zamboni et al., 2009). Depressed patients also show microstructural abnormalities in this region (Alexopoulos et al., 2008; Ma et al., 2007; Sheline et al., 2008). Finally, neuroimaging studies have implicated the precuneus in production and alteration of subjective experience (Cavanna & Trimble, 2006; Fiset et al., 1999; Lou, Nowak, & Kjaer, 2005; Vogt & Laureys, 2005). Changes in subjective experiences mediated through religious beliefs may exert long‐lasting changes in precuneus structure or connectivity, which in turn lead to resilience to depression.

Many previous studies have implicated the reliable role of frontal lobe in depressive phenotypes, onset, duration, and treatment efficacy. For example, some studies reported changes in frontal lobe are associated with problems in motivation, subjective emotional experience, and emotional expression (Stuss, Gow, & Hetherington, 1992). Dorsolateral frontal and medial frontal areas were found to be associated with religious recitation in self‐identified religious subjects (Azari et al., 2001), and the right hemisphere in particular may play an important role for elements of self that are ingrained elements of personality, such as religious values (Devinsky & Lai, 2008). We also have reported reduced frontal and parietal white matter volumes in offspring at high risk for depression (regardless of illness history), suggesting hypoplasia of frontal and parietal white matter as a potential endophenotype for familial depression (Dubin et al., 2012). In addition, white matter reductions, particularly in the superior longitudinal fasciculus tract that connects the frontal to parietal regions, were found in high‐risk subjects (Peterson & Weissman, 2011). Adults who reported high religions importance at T25 were found to have thicker cortices in frontal lobe of the right hemisphere (Miller et al., 2014). In the present analysis, we found that these differences were significantly attenuated in individuals reporting high importance of R/S beliefs. Our analyses infer that microstructure changes in fiber pathways in the frontal lobe may be attributed to the interrelationship between familial risk for depression and R/S beliefs, which agreed with those reported findings. The finding of this association thus may contribute to our understanding of the neurobiology mechanisms underpinning depression.

Our analysis also observed group differences in white matter regions close to the temporal lobe, which is known for playing an important role in emotion regulation (Dolcos, LaBar, & Cabeza, 2004; Ma et al., 2007). Numerous studies have implicated the role of middle temporal regions in clinical depression (Lorenzetti, Allen, Fornito, & Yücel, 2009) as well as in religious beliefs (Harris et al., 2009; Kapogiannis, Barbey, Su, Zamboni et al., 2009). Our EEG study reported that right‐hemispheric parieto‐temporal dysfunction, whether indexed by alpha asymmetry or cortical thinning, may serve as a familial trait marker of vulnerability to MDD (Talati, Weissman, & Hamilton, 2013). We also had previously reported persons at high risk for major depression, based on their family history, have thinner temporal cortices (Peterson et al., 2009). In addition, cortical thicknesses in the temporal areas correlate positively with local white matter volumes in the region of significant white matter hypoplasia (Dubin et al., 2012). In the present study, individuals at low risk with high importance of R/S beliefs showed reduced EAR in inferior temporal gyrus when compared with those reported low importance of R/S beliefs. Consistent with the reported findings, our results at the similar location in the temporal lobe may suggest that microstructure changes in the fiber pathways locally in the temporal lobe could be the result of the interactions between familial risk for depression and R/S beliefs, although the precise microstructural changes regarding the underlying fiber organization were not exactly known. It is worth noting that the association between temporal lobes and religious beliefs was barely reported in previous studies. In addition, because the mathematical model is relatively simple, DTI typically cannot distinguish the cases of degraded fiber organization (e.g., less fiber connectivity) and of developing more complex structures (increased fiber connectivity such as fiber crossing, fending or kissing) when EAR or FA values decrease. Therefore, interpretation of changes in measured diffusion tensor should be performed with care (Alexander, Lee, Lazar, & Field, 2007), and our findings may need further examination and verification in our further efforts to clarify the true mechanism of the changes.

Although the mechanisms underlying a potential protective effect require further investigation (see limitations), the findings in the above regions converge on a consistent pattern: where having highly important R/S beliefs minimizes brain differences otherwise associated with familial risk for depression. In other words, individuals who are at high familial risk for depression tend to adopt a neural signature that more closely resembles those at low familial risk, whether they report high importance of R/S beliefs.

It also is worthy of being noticed that R/S may change in a person's lifetime, so that the difference of one's R/S belief may have different impact on one's brain microstructure regarding possible resilient effect to developing MDD. Our previous study measured temporal stability of R/S beliefs and reported that in the 5 year between T20 and T25, among those reported importance of R/S beliefs at both time points, 12 participants were stable high as they reported consistently high importance; 53 were stable low as they reported consistently low importance; and only 17 were unstable (Miller et al., 2014). Taken together, for majority of the participants (79.27%), importance of R/S beliefs remained stable across the Waves 5 and 6. In the current study, we measured this again across Waves 5 and 6 (T25 and T30) among those 83 participants who had R/S reports in both time points no matter whether or not the participant has usable DTI data and found that 60 remained stable and 23 were unstable. The stability of R/S beliefs remained from T25 to T30 was 72.29%. However, temporal causality warrants a further investigation, as brain changes could have preceded the R/S report.

### 4.2 Limitations

The findings should be interpreted within the context of a number of limitations. First, the sample size, though larger than that of most neuroimaging studies of religiosity, is still modest, and replication in independent populations is warranted. The sample size also precluded our ability to formally test for statistical interactions between the effects of the importance of R/S beliefs and those of familial risk status on brain outcomes. Second, the R/S variable represents a single time point, and analyses do not account for potential changes in religiosity over time. The R/S variable also does not distinguish between religious versus spiritual experiences. Third, the analysis presented in this study was based purely on R/S and DTI data at T30 (Wave 6), because DTI data were only acquired at this time point. Although we have seen that the R/S beliefs of the participants were quite stable (79.27% from T20 to T25 and 72.29% from T25 to T30), excluding participants whose R/S beliefs were unstable certainly would make the analyses more reliable. We preferred using participants who reported stable R/S historically at all time points from T20 to T30. However, we found this was not possible even when only two time points, T25 and T30, were considered, due to the inadequate overlap of the availability of the imaging and R/S data. To qualify for such an analysis that demanded stable R/S beliefs across T25 and T30, a participant must meet the following three requirements simultaneously: (a) having usable DTI data; (b) having R/S measures at both time points; and (c) the R/S measures across T25 and T30 were either consistently high or consistently low. Unfortunately, excluding the participants who did not report R/S data at either time point and those reported unstable R/S beliefs from the original sample resulted in some subgroups consisting of too few participants to conduct a statistically meaningful analysis (some subgroups thus included as few as two or nine participants). Our future efforts will try to ensure that adequate participants with stable R/S beliefs will be included so that the analysis results can be more reliable. Finally, the sample is based on families of European, largely Italian, ancestry, who were also predominantly Christian. Associations may thus not readily generalize to other populations or religious denominations.

## 5 CONCLUSIONS

Most of the previous imaging studies have investigated the roles and functions of various cortical regions in the gray matter in relation to religious beliefs. Using DTI, the current study investigated white matter microstructure and integrity in relation to religious belief and correlated this with different levels of familial risk for development of MDD. The work thus provided a more comprehensive view of the brain microstructural differences related to religion and spirituality in families at high and low risk for depression.

Despite the above limitations, our findings suggest that the reported high importance of R/S beliefs may have effects on white matter integrity in the bilateral frontal lobe, temporal lobe, and parietal lobe, particularly the bilateral precuneus. While these regions are also associated with risk of developing MDD, reorganization of white matter through R/S may help protect individuals from going on to develop the illness. As future waves of data are being collected, we will be able to predictively examine whether the EAR signatures identified here protect against subsequent onset of illness among those at high familial risk for the disorder.

All of the findings aforementioned support what we expected that compared to those at low familial risk for MDD, individuals at high familial risk for MDD show significant white matter microstructural changes in white matter tracts adjacent to brain regions implicated familial risk for developing MDD. Although the mechanisms underlying the R/S beliefs’ potential protective effect require further investigation (see limitations), the findings in our analyses converge to a consistent pattern: High importance of R/S beliefs minimizes brain differences otherwise associated with familial risk for depression. In summary, individuals at high familial risk for depression typically share a neural signature that is similar to the one that can be found in those at low familial risk, as long as they take R/S beliefs as highly important.

## CONFLICT OF INTEREST

None declared.
